# Suitability of visual cues for freezing of gait in patients with idiopathic Parkinson’s disease: a case–control pilot study

**DOI:** 10.1186/s12984-023-01214-8

**Published:** 2023-07-18

**Authors:** Eui Jin An, Woo-Sob Sim, Seung Min Kim, Jun Yup Kim

**Affiliations:** 1 Department of Physical Medicine and Rehabilitation, Veterans Health Service Medical Center, Seoul, Republic of Korea; 2 Department of Prosthetics and Orthotics Center, Veterans Health Service Medical Center, Seoul, Republic of Korea; 3 Department of Neurology, Veterans Health Service Medical Center, Seoul, Republic of Korea; 4 Department of Physical Medicine and Rehabilitation, Hanyang University Medical Center, Seoul, Republic of Korea; 5 Veterans Medical Research Institute, Veterans Health Service Medical Center, Seoul, Republic of Korea; 6 Mailing address:, 222-1, Wangsimni-ro, Seongdong-gu, Seoul, 04763 Republic of Korea

**Keywords:** Idiopathic Parkinson’s disease, Freezing of gait, Visual cueing, Striatum, Putamen

## Abstract

**Background:**

Freezing of gait (FOG) is one of the most debilitating symptoms in patients with idiopathic Parkinson’s disease (IPD). Visual cues can relieve FOG symptoms. However, there is no consensus on patient characteristics that can benefit from visual cues. Therefore, we examined the differences in IPD patient characteristics according to the effectiveness of visual cueing.

**Methods:**

Through gait experiments, we investigated the number of FOG occurrences, average FOG period per episode, proportion of FOG duration in the total gait cycles, and FOG-free period gait spatiotemporal parameters in ten participants diagnosed with FOG due to IPD. Subsequently, the differences between their clinical characteristics and striatal dopamine active transporter availability from six subregions of the striatum were compared by dividing them into two groups based on the three reduction rates: occurrence numbers, mean durations per episode, and proportion of FOG duration in the total gait cycles improved by visual cueing using laser shoes. The relationships among these three reduction rates and other FOG-related parameters were also investigated using Spearman correlation analyses.

**Results:**

According to the three FOG-related reduction rates, the group assignments were the same, which was also related to the baseline self-reported FOG severity score (New Freezing of Gait Questionnaire): the more severe the FOG, the poorer the response to the visual cueing. By visual cueing, the better response group demonstrated the characteristics of lower new FOG questionnaire total scores, higher dopamine active transporter availability of the anterior and posterior putamen, and shorter mean duration of FOG per episode in the absence of cueing. These results were replicated using Spearman correlation analyses.

**Conclusions:**

For FOG symptoms following IPD, gait assistance by visual cueing may be more effective when the total NFOGQ score is lower and the DAT of putamen is higher. Through this study, we demonstrated clinical and striatal dopaminergic conditions to select patients who may be more likely to benefit from visual cueing with laser shoes, and these findings lead to the need for early diagnosis of FOG in patients with IPD.

**Trial registration:**

ClinicalTrials.gov identifier: NCT05080413. Registered on September 14, 2021.

**Supplementary Information:**

The online version contains supplementary material available at 10.1186/s12984-023-01214-8.

## Background

Although the pathophysiology of idiopathic Parkinson’s disease (IPD) remains unclear, it is known that the loss of part of the dopaminergic neurons in the substantia nigra results in a specific disorganization of basal ganglia circuits [1, 2]. Loss of dopaminergic and/or cholinergic neurons leads to motor dysfunction, which is often accompanied by a characteristic gait disorder called freezing of gait (FOG) [3–5]. FOG is defined as a “brief, episodic absence or marked reduction of forward progression of the feet despite the intention to walk” which in turn increases the fear of walking and the risk of falls [6, 7]. Most of these episodes occur when participants initiate a gait or attempt to turn around [8, 9], and the symptoms can also be aggravated when faced with a narrow corridor or emotional stress [10, 11].

Clinically, FOG is one of the biggest factors that reduce quality of life due to fall-related injuries and fear of falling in patients with IPD. A large-scale study of 6,620 IPD patients has identified that nearly 50% of patients experienced freezing regularly and 80% experienced freezing at an advanced stage of the disease [12]. Despite its clinical importance and considerable frequency of occurrence, FOG is considered one of the most debilitating yet the least understood symptoms of IPD.

Previous studies have reported that visual cues can alleviate FOG symptoms and reduce the number and duration of freezing episodes [13, 14]. Visual cues using laser shoes demonstrated significant reduction in the number of FOG episodes in both the “off” and “on” phases [15].

Although the effect of visual cueing on FOG has been widely studied, there is no consensus on the characteristics of the target subpopulation that can achieve a larger effect or the neural mechanisms underlying the acute effect of visual cueing. In addition, several studies have reported a relationship between FOG symptoms and striatal subregional patterns of dopamine active transporter availability (DAT) loss, but no studies have examined the relationship of visual cueing effectiveness and striatal subregional DAT [16, 17]. Thus, we hypothesized that clinical variables reflecting the progression of IPD and the severity of FOG would be related to the magnitude of FOG symptom reduction with visual cueing using laser shoes, and that the magnitude would be also related to decreased dopaminergic metabolism at specific neuroanatomical locations in the striatum. This case–control study aimed to define the characteristics of participants with IPD in which FOG symptoms can be more effectively alleviated by visual cueing.

## Methods

### Participants

This study was conducted in accordance with the Strengthening the Reporting of Observational Studies in Epidemiology statement [18]. To identify the participant characteristics in which visual cues work effectively for alleviation of freezing episodes, participants diagnosed with IPD and those suffering from FOG were screened for study inclusion.

The inclusion criteria were as follows: written informed consent; diagnosis of IPD according to the UK Brain Bank Guidelines [19]; diagnosed with FOG according to the New Freezing of Gait Questionnaire (NFOGQ) and Movement Disorder Society-sponsored revision of the Unified Parkinson’s Disease Rating Scale (UPDRS) [20, 21]; underwent ^18^ F-fluoro-propyl-carbomethoxy-iodophenyl-tropane (FP-CIT) positron emission tomography (PET); underwent a 3-Tesla T_1_-weighted MRI scan; with < 1-month interval between PET and gait experiment; scored ≥ 24 points in the Korean version of the Mini-Mental Status Examination (K-MMSE) [22]; and independent gait for at least 100-meter without a walking assistance device. The exclusion criteria were as follows: diagnosis of brain lesions other than IPD, such as traumatic brain injury or stroke; evidence of old cerebral lesions > 3 mm in diameter on MRI [23]; history of major trauma or surgery of the lower extremities that can affect the gait function; impaired visual and/or hearing function; and with gait disturbance from causes other than IPD.

### Demographic and clinical data acquisition

The following demographic and clinical data were collected from the electronic medical records: age, sex, disease duration (length of time from the first diagnosis of IPD to the date of the experiment), and the Digital Imaging and Communications in Medicine files of MRI and ^18^ F-FP-CIT PET.

The following information was evaluated in the experimental stage through the participants: K-MMSE score, Hoehn & Yahr (HY) stage, UPDRS part III total score (UPDRS-III), NFOGQ total score, and total numbers and mean durations of FOG episodes according to each of the three experimental conditions.

### Apparatus

**Fig. 1:**
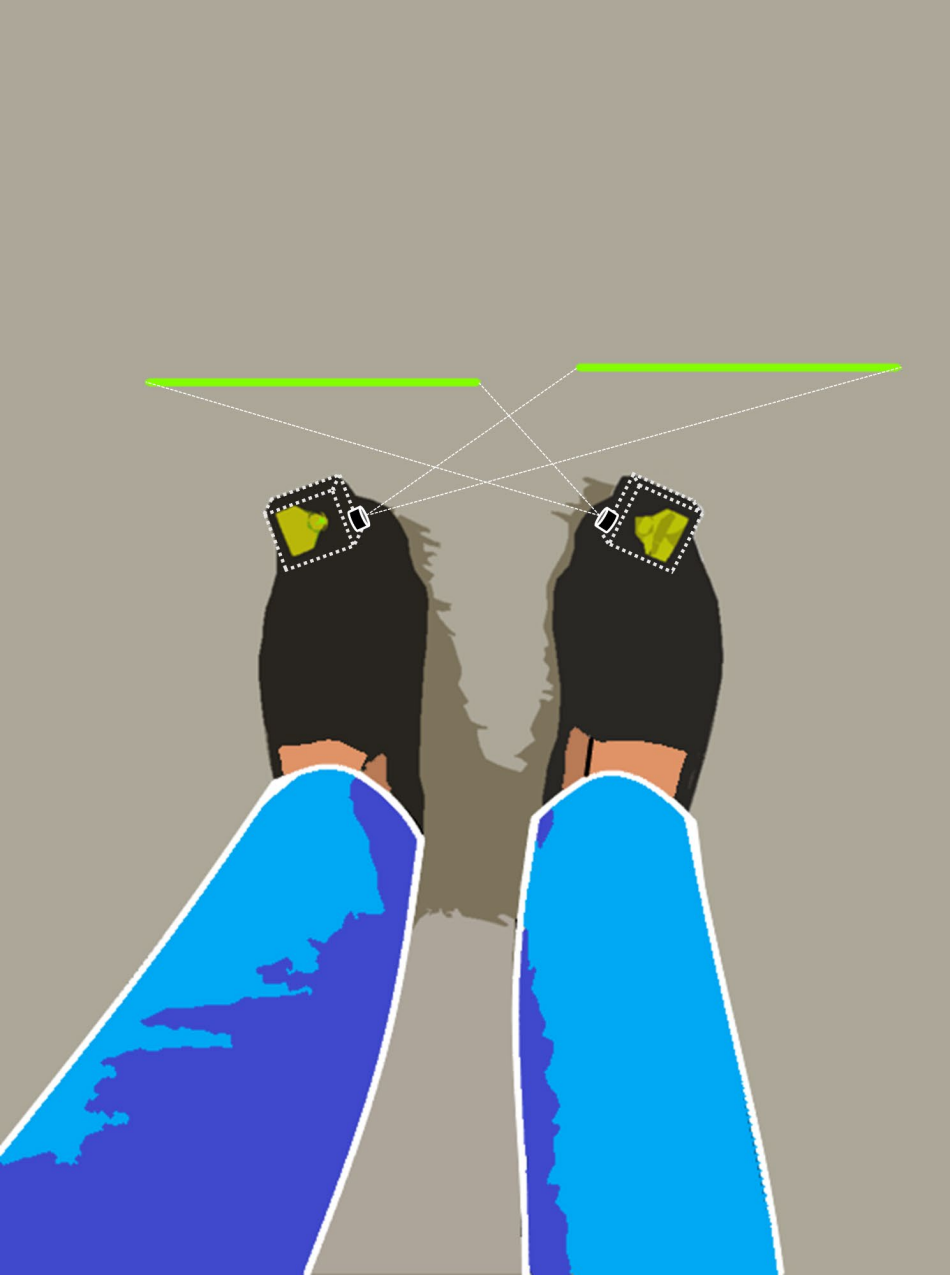
Illustration of the experimental setup for the visual cueing with laser-beam

To minimize the effect of external noise, the experimental site was selected as a sound-insulated, flat area with a plain grey-colored floor. As shown in Fig. 1, the PathFinder system (WalkWithPath Ltd, Denmark) was worn on the shoes for visual cueing. This device plays the role of visual cueing to cross the line by firing a green laser line on the ground in front of the foot on the opposite side of the dorsum of each foot. After the laser device was mounted on the shoe, the direction of the laser firing nozzle was adjusted so that the laser beam was drawn parallel to the coronal plane of the subject on the ground 5 cm in front of the opposite foot.

### Experimental protocol

To minimize the effects of anti-Parkinson’s disease medications on FOG symptoms, all participants were evaluated and tested in the “off” state at least 12 h after the last anti-Parkinson’s disease medication administration [24]. All participants were given 10 min to familiarize themselves with the visual cues in order to dispel any biases that may arise from unfamiliarity. The laser shoes continuously emitted lasers, and at the loading response of the foot in its stance phase, the green laser appeared on the ground and drew a fixed line from the pre-swing phase to the terminal swing in front of the participant’s foot opposite the laser shoe. Participants were instructed to walk with the idea of crossing the green line drawn on the ground with the foot in its swing phase. Finally, they were asked to walk three laps on a 10-meter track in the observation laboratory, under each of the following two conditions: without cueing and with visual cueing. Videos (30 frames-per second) containing all cycles of gait were recorded to evaluate the exact number and duration of FOG episodes.

**Fig. 2:**
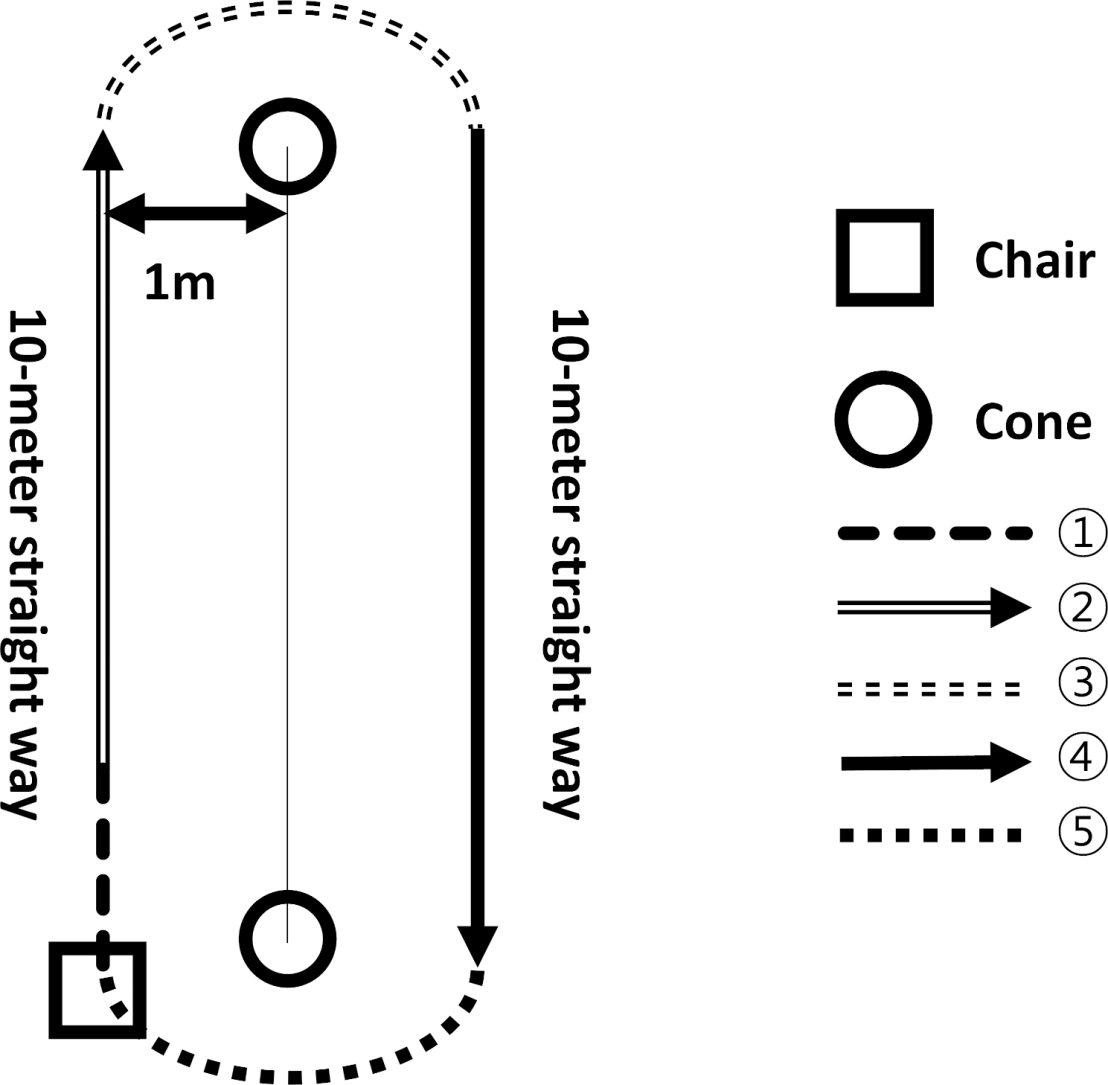
Schematic diagram of gait experiments

After the abovementioned preparations were completed, the participants proceeded with the experiment using the following procedure (Fig. 2). For each of the two conditions, participants repeated the following gait tasks for 3 times for each condition (totaling to six laps per participant): first, when the start signal is received, the participant gets up from the chair and begins walking (initiation task); second, walks 10 m straight; third, walks around distal cone (turning task); fourth, walks 10 m straight; and last, walks around the proximal cone (turning task) and sits down on a chair. To exclude the effect of accumulating fatigue due to walking, a 10-minute break was given between each condition.

During the gait of the three laps for each condition, the following FOG-related parameters in the first, third, and fifth sections of the gait tasks (gait initiation, and the two turning conditions), which are phases wherein FOG frequently occurs, were assessed by two rehabilitation medicine physicians specializing in gait analysis.: total occurrences, mean duration of each episode, total duration of three laps, and total duration of FOG episodes within three laps. The video frames with start and termination of FOG episodes were defined by the previous publications: “a sudden increase in stepping frequency, reduction of step height, and motor block as recognizable by the negligible step amplitude preceded by the premature lifting of the heel” [10, 25]. In addition, to determine the effect of visual cueing on FOG-free gait cycles, the spatiotemporal parameters of gait (gait speed, cadence, single/dual-limb support duration ratio) during the gait cycles without FOG were analyzed frame-by-frame using the ImageJ program, version JAVA 1.53k (National Institutes of Health, USA) [26].

### Acquisition and preprocessing of ^18^ F-FP-CIT PET images

All participants fasted for at least 12 h and discontinued all anti-Parkinson’s disease medications for at least 6 h prior to the ^18^ F-FP-CIT PET scan [27, 28]. The participants were intravenously injected with 3.7 MBq/kg of ^18^ F-FP-CIT, and PET images were obtained for 20 min using Discovery STE (GE Healthcare, Milwaukee, WI, USA) 2 h after the injection [28, 29]. For each participant, the reconstructed PET images were co-registered with the 3T T1-weighted-MRI and normalized to the Montreal Neurological Institute-152 (MNI152) space (2 × 2 × 2 mm^3^ per voxel).

### Quantitative analyses of the ^18^ F-FP-CIT PET images

Based on the anterior commissure coronal plane of the Oxford-Imanova Striatal Structural Atlas, the caudate and putamen were divided into anterior and posterior portions, respectively. The boundary between the posterior and ventral putamen was at the anterior–posterior commissure transaxial plane [16]. Finally, six volumes of interest (VOIs) were segmented (anterior/posterior caudate, anterior/posterior/ventral putamen, and ventral striatum).

The calcarine fissure and surrounding cortex were selected from the Automated Anatomical Labeling atlas as a reference region for calculating the DAT [16, 30]. The standardized uptake value ratio (SUVR), as a DAT surrogate for each VOI was calculated using the following equation: mean uptake value of the VOI / mean uptake value of the calcarine fissure and surrounding cortex.

### Group assignment for case?control analysis

Participants were divided into better and poorer response groups according to three criteria of the reduction rate as follows: the occurrence reduction rate (RRO) = (total number of occurrences of FOG episodes in non-cueing - total number of occurrences of FOG episodes in cueing) / total number of occurrences of FOG episodes in non-cueing; the mean reduction rate of duration (RRD) = (mean duration of FOG episodes in non-cueing - mean duration of FOG episodes in cueing) / mean duration of FOG episodes in non-cueing; and the reduction rate of proportion of FOG duration in the total gait cycles (RRP) = [(total duration of FOG episodes within three laps / total duration of three laps) in non-cueing – (total duration of FOG episodes within three laps / total duration of three laps) in cueing] / (total duration of FOG episodes within three laps / total duration of three laps) in non-cueing. To identify the factors associated with better responsiveness, the demographic and clinical characteristics and DAT between the two groups were compared.

### Correlation analyses of FOG-related parameters and SUVRs

Since variables such as RRO, RRD, and RRP have some overlap between the two groups and cannot be judged by a strictly bimodal distribution, we also checked for nonparametric correlations between each variable. Therefore, unadjusted Spearman correlation analyses were performed using FOG-related parameters (i.e., disease duration, HY stage, UPDRS-III, NFOGQ total score, RRO, RRD, and RRP) and SUVRs for each of the six VOIs.

### Statistical analysis

Due to the small number of participants in this pilot study, all statistical analyses were performed using non-parametric methods. Demographic and clinical data are presented as median (25th, 75th percentiles) or number, depending on the type of distribution. Between-group comparisons of age, reduction rate, DAT, K-MMSE score, disease duration, UPDRS-III, HY stage, and NFOGQ total score were statistically tested using the Mann–Whitney U-test, and sex using Fisher’s exact test. The Wilcoxon signed-rank test was performed to test the statistical significance of the effects of visual cueing within groups. Two-tailed *p*-values < 0.05 were considered significant. The effect size Cohen’s r was calculated for significant comparisons to examine the levels of change [31]. All data analyses were performed using the R Statistical Package, version 3.6.1 (R Foundation for Statistical Computing, Vienna, Austria). Image preprocessing was performed using Statistical Parametric Mapping 12 (Wellcome Department of Imaging Neuroscience, Institute of Neurology, University College London), and further analyses of PET images were implemented using in-house MATLAB (R2020b, The MathWorks Inc.) scripts. The power estimation required for further studies was also performed, which was calculated using G*Power, version 3.1.9.7 [32]. Mean and standard deviation values of striatal DAT were used to provide at least power of 95% with the following parameters: probability of type I error 0.05, and effect sizes from Cohen’s effect size d [31].

### Ethics statement

This study was approved by the Institutional Review Board of the Veterans Health Service Medical Center, and written informed consent in accordance with the institutional review board was obtained from all participants before enrollment. The clinical trial was registered as NCT05080413.

## Results

### Baseline characteristics of participants and group assignments

Overall, 11 participants diagnosed with IPD and suffering from FOG were screened for inclusion, and one was excluded from the study because the K-MMSE did not reach a score of 24. Finally, the remaining ten participants were included for analysis. The participants’ characteristics are presented in Table 1. Of the ten participants, only one was female, the disease duration ranged from 1.5 to 10 years, and the age of the participants at the time of the experiment ranged from 69 to 88 years. The participants’ UPDRS-III ranged from 11 to 64, and for the HY stage, only one participant was in stage 2, and the remaining nine were in stage 3. Based on RRO, RRD, and RRP, the median values of all participants were 0.33, 0.19, and 4.95, respectively, and when divided into two groups based on the three criteria, the same group assignment was determined.

**Table 1:** Participant characteristics

	Total (n = 10)	Better response group (n = 5)	Poorer response group (n = 5)	Between group difference *p*-value, effect size r
**Sex, male / female**	9 / 1	4 / 1	5 / 0	1.000
**Age**	73.50 (72.00, 80.00)	72.00 (70.50, 84.00)	74.00 (73.00, 77.50)	0.310
**Disease duration, years**	4.50 (2.00, 6.25)	4.00 (2.25, 7.50)	5.00 (2.00, 7.50)	0.841
**HY stage**	3.00 (3.00, 3.00)	3.00 (2.50, 3.00)	3.00 (3.00, 3.00)	0.690
**UPDRS-III**	36.00 (14.00, 49.75)	15.00 (11.00, 39.00)	45.00 (31.00, 62.50)	0.095
**NFOGQ**	26.50 (21.75, 28.00)	22.00 (17.50, 24.50)	28.00 (27.50, 28.00)	0.008, − 0.852*
**K-MMSE**	26.50 (24.00, 28.00)	28.00 (24.50, 29.00)	26.00 (23.50, 27.50)	0.310
**RRO**	0.33 (0.19, 0.69)	0.67 (0.50, 0.83)	0.22 (0.06, 0.28)	0.008, − 0.800*
**RRD**	0.19 (-0.08, 0.48)	0.48 (0.38, 0.54)	−0.06 (-0.15, 0.02)	0.008, − 0.826*
**RRP, %**	4.95 (-10.13, 15.40)	15.38 (9.43, 20.09)	−8.36 (-15.47, 2.54)	0.008, -0.826*

Data are presented as median (25th, 75th percentiles), or number. *HY* Hoehn & Yahr, *UPDRS-III* part III total score of the Movement Disorder Society-sponsored revision of the Unified Parkinson’s Disease Rating Scale, *NFOGQ* New Freezing Of Gait Questionnaire, *K-MMSE* Korean version of Mini-Mental Status Examination, *RRO* reduction rate of occurrence, *RRD* reduction rate of mean duration, *RRP* reduction rate of FOG proportion in the total gait cycles.

*Significant difference with Mann–Whitney U-test.

### FOG-free period spatiotemporal gait parameters

There were no statistically significant differences between the two groups in spatiotemporal gait parameters in the FOG-free period with or without visual cues (Additional file 1: Table S1).

### Within group differences

Of the two groups, only the better response group demonstrated a significant improvement in the number of FOG episodes, mean FOG duration, and proportion of FOG duration in the total gait cycles by visual cueing (Table 2). Furthermore, also in non-responders, the number of FOG episodes showed decrements with visual cueing compared with non-cueing condition, although statistically not significant (*p* = 0.07). Meanwhile, the poorer response group demonstrated an increased median value of average FOG duration (*p* = 0.27) and proportion of FOG duration (*p* = 0.23) with visual cueing, although statistically not significant.

### Between group differences

Sex, age, disease duration, HY stage, UPDRS-III and K-MMSE scores were not significantly different between both groups (Table 1). The difference in the UPDRS-III was not significantly different between both groups (*p* = 0.095); however, the median values were 15.0% and 45.0% in both groups, respectively, indicating a relatively higher severity of IPD in the poorer response group. The NFOGQ total score was significantly different between both groups (*p* = 0.008, r = − 0.852), and the median values of each group were 22.0% and 28.0%, respectively.

**Fig. 3:**
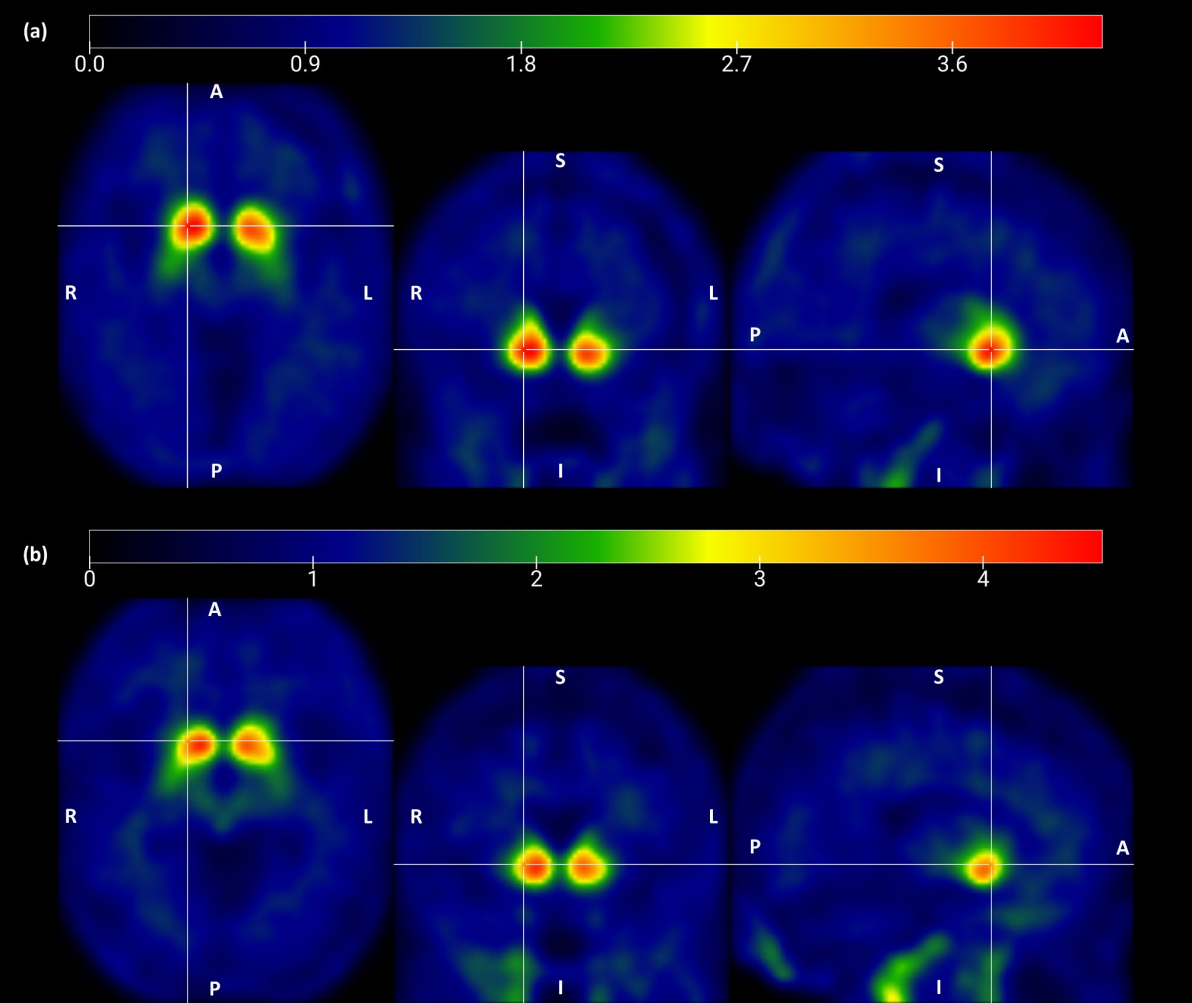
Overlaid map of FP-CIT standardized uptake value ratios from the **(a)** better response and **(b)** poorer response groups. For visualization purposes, overlaid maps were spatially smoothed using a Gaussian kernel of 8-mm full-width at half maximum. The coordinate indicated by the crosshair is the anterior putamen, which are positions corresponding to 10, 10, -5 in the MNI-152 coordinate system. The color bar represents the uptake intensity from the minimum to the maximum (scaled from 0 to maximum values). *A* anterior, *P* posterior, *R* right, *L* left, *S* superior, *I* inferior, *MNI* Montreal Neurological Institute

Table 2 summarizes the comparison of outcomes between both groups. In terms of DAT, the VOIs that had a significant difference between the two groups were anterior (*p* = 0.009, r = − 0.826) and posterior putamen (*p* = 0.047, r = − 0.628). In these two VOIs, the better response group demonstrated a greater uptake of FP-CIT. In other striatal VOIs, no significant differences in DAT were observed between the two groups. Figure 3 depicts the overlays of SUVR of FP-CIT in each of the two groups, indicating that the higher uptake area of the anterior putamen is broader in the better response group.

FOG episodes occurred in all nine possible phases (three laps) in all ten participants in the absence of cueing (Table 2). With visual cueing, a significant difference was observed between the two groups (*p* = 0.011, r = − 0.800), and the median numbers of both groups were 3 and 7, respectively. The mean duration of FOG episodes was significantly different between the two groups, with or without visual cues. The difference in mean duration of FOG episodes between the two groups according to visual cueing increased further, with *p*-values of 0.028 and 0.009, and effect sizes of − 0.694 and − 0.828, respectively. For the proportion of FOG duration in the total gait cycles, there was a significant difference between the two groups, only with visual cueing (p = 0.008, r = − 0.826). FOG-related raw values (total number, mean duration, and proportion of duration in the total gait cycles) from each participant with or without visual cueing are presented in Additional file 1: Table S2.

**Table 2:** Comparison of characteristics between the better and poorer response groups

	Better response group (n = 5)	Poorer response group (n = 5)	Between group difference *p*-value, effect size r
**DAT SUVR**			
**Anterior putamen**	2.86 (2.47, 3.11)	1.86 (1.84, 1.92)	0.009, − 0.826*
**Posterior putamen**	1.51 (1.44, 1.66)	1.40 (1.35, 1.41)	0.047, − 0.628*
**Ventral putamen**	1.82 (1.68, 2.05)	1.86 (1.57, 1.97)	0.917
**Anterior caudate**	1.66 (1.09, 2.45)	2.12 (1.60, 2.38)	0.465
**Posterior caudate**	1.02 (0.47, 1.36)	1.39 (0.94, 1.62)	0.117
**Ventral striatum**	3.68 (3.50, 4.27)	3.85 (3.81, 3.94)	0.117
**Total occurrence number of FOG**			
**Without cueing**	9.00 (9.00, 9.00)	9.00 (9.00, 9.00)	1.000
**Visual cueing**	3.00 (1.50, 4.50)	7.00 (6.50, 8.50)	0.011, − 0.800*
**Within group difference**, ***p*** **-value, effect size r**	0.04, − 0.91 **	0.07	NA
**Mean duration of FOG**			
**Without cueing, seconds**	4.10 (3.65, 5.00)	7.10 (5.15, 17.00)	0.028, − 0.694*
**Visual cueing, seconds**	2.20 (1.95, 2.65)	8.20 (5.30, 18.30)	0.009, − 0.828*
**Within group difference**, ***p*** **-value, effect size r**	0.04, − 0.91 **	0.27	NA
**Proportion of FOG duration in the total gait cycles**			
**Without cueing, %**	50.00 (48.57, 50.46)	50.52 (48.88, 63.51)	0.220
**Visual cueing, %**	43.06 (38.90, 45.28)	57.86 (54.89, 61.92)	0.008, − 0.826*
**Within group difference**, ***p*** **-value, effect size r**	0.04, − 0.90**	0.23	NA

Data are presented as median (25th, 75th percentiles), or number. *DAT* dopamine active transporter availability, *SUVR* standardized uptake value ratio, *FOG* freezing of gait, *NA* not applicable.

*Significant difference in the Mann–Whitney U-test *p*-value (effect size).

**Significant difference in the Wilcoxon signed rank test.

### Correlation among FOG-related parameters and SUVRs

**Fig. 4:**
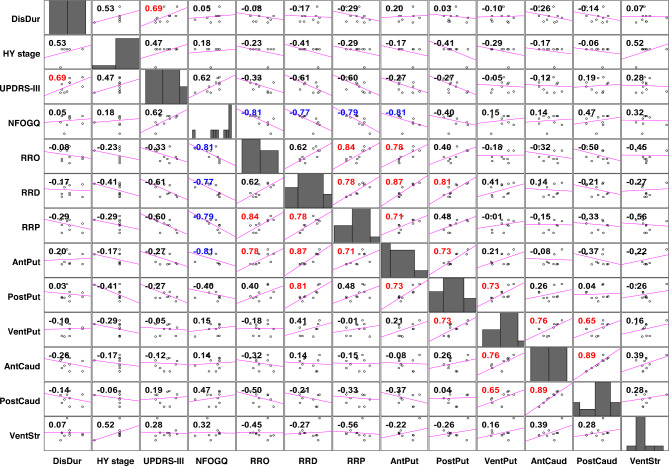
Correlation matrix plot showing inter-variable unadjusted Spearman correlations and distribution of freezing of gait-related parameters. The numbers on the matrix plot show Spearman’s rho. Red and blue numbers indicate statistically significant positive and negative rho values, respectively. *DisDur* disease duration, *HY* Hoehn & Yahr, *UPDRS-III* part III total score of Movement Disorder Society-sponsored revision of the Unified Parkinson’s Disease Rating Scale, *NFOGQ* New Freezing of Gait Questionnaire total score, *RRO* occurrence reduction rate, *RRD* mean reduction rate of duration, *RRP* reduction rate of proportion of FOG duration in the total gait cycles, *AntPut* Anterior putamen, *PostPut* Posterior putamen, *VentPut* Ventral putamen, *AntCaud* Anterior caudate, *PostCaud* Posterior caudate, *VentStr* Ventral striatum

Figure 4 shows the correlations and distribution among FOG-related parameters and SUVRs for each of the six VOIs. RRO, RRD, and RRP were all significantly negatively correlated with NFOGQ total score, and these three variables were significantly positively correlated with each other in all correspondences except for the RRO to RRP relationship. In addition, all three variables were significantly positively correlated with DAT SUVR in the anterior putamen, and RRD was significantly positively correlated with DAT SUVR in the posterior putamen. Within FOG-related parameters, there was a statistically significant positive correlation between disease duration and UPDRS-III. In addition, NFOGQ total score was significantly negatively correlated with SUVR in the anterior putamen, and some significant positive correlations were found between SUVRs across VOIs.

### Power estimation for further studies

Sample size calculations for further studies showed that 95% power for effect sizes for the anterior and posterior putamen would require a total sample size of 10 (Cohen’s effect size d = 2.87) and 20 (Cohen’s effect size d = 1.79) participants, respectively.

## Discussion

In this study, we demonstrated for the first time that patients with IPD with FOG who have lower NFOGQ total scores, higher DAT of the anterior and posterior putamen, and shorter mean duration of FOG episodes in the absence of cueing can be better candidates for reducing FOG frequency, mean duration and proportion of duration in the total gait cycles through assistance of visual cueing.

Significant difference (*p* = 0.008) in NFOGQ total scores with an effect size of − 0.852 between the two groups suggest that visual cues may be more effective in patients with lower NFOGQ total scores. The NFOGQ is a self-reported questionnaire assessing the frequency and duration of freezing episodes and its impact on the quality of life in the last month of each patient [33, 34]. Although a recent study has reported that NFOGQ is insufficient or less responsive for detecting small effect sizes, making it unsuitable as a primary outcome [35], it is still a representative evaluation index that forms a consensus in that it represents FOG severity well [36]. This study’s results revealed that it would be more effective to use visual cueing before FOG progresses to a more severe condition. Also, although not statistically significant (p = 0.095), participants in the better response group (median 15.00) showed relatively lower UPDRS-III than those in the poor response group (median 45.00). This statistical insignificance may have come from a relatively small number of participants.

Regarding spatiotemporal parameters, it has been reported that visual cueing had an effect on parts other than FOG such as increase in step / stride length, gait speed, and arm swing and decrease in cadence in previous studies [37]; however, since these studies did not analyze only FOG-free periods, it can be interpreted that the effect of visual cueing on FOG was mainly reflected. Further, in our study, gait speed, cadence, and single / double limbs support duration ratio in the FOG-free periods did not show statistically significant differences according to visual cueing or visual cueing responsiveness. Our findings suggest that visual cues primarily influence the gait periods with FOG, but reconfirmation with additional studies involving larger numbers of participants is needed.

Significant differences in both the DAT of the anterior and posterior putamen were observed between the two groups. In particular, the effect size of the DAT of the anterior putamen was larger than that of the posterior putamen. In the correlation analyses, as in the between-group analyses, higher DAT in the anterior putamen was associated with a higher effect of visual stimulation on FOG-related parameters. The posterior putamen is the main neurodegeneration target of IPD, which is related to main motor symptoms, including tremor, rigidity, and akinesia; however, as the disease progresses, the anterior putamen is also affected [38]. FOG in IPD is associated with disease severity and longer levodopa treatment [12, 39], the effect of visual cueing may be reduced in neurodegeneration that has already involved the anterior putamen. According to one study dealing with cortico-striatal connectivity in IPD patients, the changes in cortico-striatal connectivity did not spread to the visual aspect, and this observation fits with the heavy reliance of patients with IPD on sensory modalities to guide their movements [40]. With these pathogenetic characteristics, visual cueing is effective as a method to compensate for FOG symptoms using a conserved sensory function; however, the compensatory effect might diminish with disease progression.

Since the poorer response group had a significantly larger NFOGQ total score, this group could have a higher number of FOG occurrences and a longer duration of FOG compared to the other group in the absence of intervention. Moreover, in this group, even when visual cueing was used, the FOG-related dependent variables did not indicate significant improvements. Rather, the median value of the mean duration of FOG and proportion of FOG duration in the total gait cycles increased with visual cueing in the poorer response group, suggesting that visual cueing using laser shoes is likely to be less effective in patients with IPD with high initial FOG severity.

This study has several limitations. First, the number of samples was small as this was a pilot study. Although only non-parametric tests were performed for this reason, a larger number of participants with IPD with FOG with more diverse characteristics will be needed in the future to produce robust statistical inferences. Second, while it has been reported that it is often difficult to induce FOG in the clinic or gait laboratory [41, 42], all participants enrolled in this study demonstrated FOG in all possible phases in the absence of cueing. Considering this, we cannot rule out a selection bias in which only participants with relatively high FOG severity were enrolled. Therefore, it would be desirable to design a study that included participants with lower FOG severity in the future. Third, although a large-scale high-quality meta-analysis study has reported that sex was not a predictor of FOG, our study included only one female participant [43]. Fourth, since the experiment was performed only on off-state visual cues, we could not analyze patient suitability in the on-state or auditory cues. Further, although all participants were at off-state, the levodopa equivalence daily dose was not calculated resulting in possibility of bias from levodopa dose taken. Fifth, since visual cueing was provided as an auxiliary tool rather than a therapeutic one, a study to define a group with good therapeutic effects based on the effect before and after treatment by receiving gait rehabilitation treatment using visual cueing for a certain period of time will be helpful for defining the therapeutic patient suitability. Sixth, a 10-minute rest period was given between each gait condition, but all participants performed the gait task in the order of ‘visual cueing after non-cueing’, and there may have been a bias due to fatigue or carryover effect of gait training. Therefore, future studies applying random order of gait condition will be needed. Seventh, inconsistent duration of discontinuation of antiparkinsonian medication before the PET scan (6 h) and the gait test (12 h) is another limitation of this study. This inconsistency might have obscured the analysis of the relationship between FOG symptoms and dopaminergic activity. Lastly, the spatial parameters of gait (step length, etc.) could not be analyzed because the gait was recorded without a marker or infrared camera and the floor was a flat grey-colored area without any markings.

## Conclusions

The effect of visual cues was higher when FOG severity in participants with IPD was relatively lower and DAT in the putamen was relatively higher on FP-CIT PET. In particular, it was revealed for the first time that the DAT of the anterior putamen can be a predictor of the effectiveness of gait assistance through visual cueing. To help alleviate FOG symptoms through visual cues, it is important to sensitively identify the presence of FOG symptoms before FOG or IPD progresses, and to apply visual cues to the patient early to reduce fear of injury or falls.

## Electronic supplementary material

Below is the link to the electronic supplementary material.

Additional File 1: A document file with additional tables.

## Data Availability

The datasets generated and analyzed are available from the corresponding author on reasonable request.

## Abbreviations

DAT: dopamine active transporter availability
FOG: Freezing of gait
FP-CIT: ^18^ F-fluoro-propyl-carbomethoxy-iodophenyl-tropane
HY: Hoehn & Yahr
IPD: Idiopathic Parkinson’s disease
K-MMSE: Korean version of Mini-Mental Status Examination
UPDRS: Movement Disorder Society-sponsored revision of the Unified Parkinson’s Disease Rating Scale
MNI152: Montreal Neurological Institute-152
MRI: magnetic resonance imaging
NFOGQ: New Freezing of Gait Questionnaire
PET: positron emission tomography
RRD: reduction rate of mean duration
RRO: occurrence reduction rate
RRP: reduction rate proportion in the total gait cycles
SUVR: standardized uptake value ratio
VOI: volume of interest
