# Supplementary material for: Suitability of visual cues for freezing of gait in patients with idiopathic Parkinson’s disease: a case–control pilot study
Source: J Neuroeng Rehabil. 2023 Jul 18;20:91. doi: 10.1186/s12984-023-01214-8 (PMC10354967; doi:10.1186/s12984-023-01214-8)
Supplement: Supplementary file 1 — Additional File 1: A document file with additional tables. [file 12984_2023_1214_MOESM1_ESM.docx]

**Additional file 1**

Table S1. Comparison of spatiotemporal gait parameters in FOG-free periods between the better and poorer response groups

|  | **Better response group (n = 5)** | **Poorer response group (n = 5)** | **Between group difference**  ***p*-value, effect size r** |
| --- | --- | --- | --- |
| **Gait Speed, m/s** |  |  |  |
| **Without cueing** | 1.31 (1.18, 1.41) | 1.18 (1.12, 1.26) | 0.151 |
| **Visual cueing** | 1.38 (1.16, 1.47) | 1.18 (1.14, 1.26) | 0.310 |
| **Within group difference, *p*-value, effect size r** | 0.68 | 0.50 | NA |
| **Cadence, cycle/min** |  |  |  |
| **Without cueing** | 124.00 (113.00, 132.00) | 121.00 (117.00, 129.50) | 0.841 |
| **Visual cueing** | 119.00 (113.00, 134.00) | 123.00 (120.00, 127.50) | 0.690 |
| **Within group difference, *p*-value, effect size r** | 1.00 | 0.49 | NA |
| **Single / double limbs support duration ratio** |  |  |  |
| **Without cueing** | 1.95 (1.87, 2.13) | 1.81 (1.75, 1.94) | 0.095 |
| **Visual cueing** | 2.08 (1.90, 2.10) | 1.91 (1.73, 1.96) | 0.095 |
| **Within group difference, *p*-value, effect size r** | 0.69 | 0.89 | NA |

Data are presented as median (25th, 75th percentiles). *FOG,* freezing of gait; *NA,* not applicable.

Table S2. Raw values of FOG-related parameters for each participant in each condition

| **Participant No.** | **Total number** | | **Mean duration, secs** | | **Proportion of duration in the total gait cycles, %** | |
| --- | --- | --- | --- | --- | --- | --- |
|  | **Without cueing** | **Visual cueing** | **Without cueing** | **Visual cueing** | **Without cueing** | **Visual cueing** |
| **Better response group** | | | | | | |
| **1** | 9 | 3 | 4.1 | 1.8 | 50 | 44 |
| **2** | 9 | 2 | 3.3 | 2.2 | 47 | 35 |
| **3** | 9 | 3 | 4.5 | 2.2 | 50 | 42 |
| **4** | 9 | 6 | 4.0 | 2.1 | 50 | 47 |
| **5** | 9 | 1 | 5.5 | 3.1 | 51 | 43 |
| **Poorer response group** | | | | | | |
| **1** | 9 | 7 | 5.4 | 5.4 | 60 | 58 |
| **2** | 9 | 9 | 12.8 | 12.3 | 67 | 66 |
| **3** | 9 | 7 | 7.1 | 8.2 | 48 | 55 |
| **4** | 9 | 6 | 4.9 | 5.2 | 51 | 55 |
| **5** | 9 | 8 | 21.2 | 24.3 | 50 | 58 |

*FOG,* freezing of gait.
